# Unresolved Crisis in Medical Education

**DOI:** 10.1155/S1064744994000451

**Published:** 1994

**Authors:** Gilles R.G. Monif, Matthew J. Severin

**Affiliations:** 1 Department of Obstetrics and Gynecology Creighton University School of Medicine 601 North 30th Street, Suite 4810 Omaha NE 68131 USA; 2 Department of Preventive Medicine and Public Health Creighton University School of Medicine Omaha NE USA

## Abstract

A crisis exists in medical education. Changes in methodology have diverted attention from synthesis to mass accumulation of factual data. The response to this crisis has been largely focused on a shell game involving new pathways and curriculum changes without addressing the critical issue of what constitutes education. The ultimate problem in medical education is a crisis of leadership. Until education is given a priority status and the obligations to teach on the part of medical educators and to learn on the part of students are translated into a creative policy by those who can lead, the wheels of learning will continue to spin without significant progress.


Infectious Diseases in Obstetrics and Gynecology 2:91–94 (1994)
© 1994 Wiley-Liss, Inc.

### Unresolved Crisis in Medical Education

**Gilles R.G. Monif and Matthew J. Severin**

*Departments of Obstetrics and Gynecology (G.R.G.M.) and Preventive Medicine and Public Health (M.J.S.), Creighton University School of Medicine, Omaha, NE*

Abstract

**A crisis exists in medical education. Changes in methodology have diverted attention from synthesis to mass accumulation of factual data. The response to this crisis has been largely focused on a shell game involving new pathways and curriculum changes without addressing the critical issue of what constitutes education. The ultimate problem in medical education is a crisis of leadership. Until education is given a priority status and the obligations to teach on the part of medical educators and to learn on the part of students are translated into a creative policy by those who can lead, the wheels of learning will continue to spin without significant progress.** © 1994 Wiley-Liss, Inc.

Key words

Medical curriculum, medical educators, medical schools, medical students

In 1865, Claude Bernard wrote his classic monograph "Introduction to the Study of Experimental Medicine." This treatise outlined the methodology for the study of medicine through the use of the scientific method. Subsequently, the tenets of this methodology have permeated the foundations of medicine. Scientists, healers, and educators have tended to disregard the lessons of nature and have become overly dependent on the gospel of the scientific approach to solve problems in modern times. This reliance on the scientific method has engendered a distrust for that which is not quantifiable. Unfortunately, the scientific method does not readily lend itself to the study of isolated occurrences or observations in nature. Many aspects of medical education lend themselves to quantification; however, they may not measure the process of learning achieved by the students. Too often, the medical student is evaluated on the basis of a preset numerical standard and not against the body of knowledge to which he or she has been exposed.

### THE PROBLEM

Tosteson wrote, "At most medical schools, general medical education is a marginal product."[1] The reasons for this are debatable and many theories have been offered. In the post-World-War-II medical-school administration circles and, to a certain extent, in academia, the perception that significant institutional growth could be achieved through the capture of federal dollars became the rule. Many institutions attained substantial internal growth by recruiting faculty members who had focused talent in analysis and explanation of biological phenomenon through experimentation. Academic advancement became progressively equated with credentialing through grantsmanship and publication success rather than through the more-difficult-to-access ability to impart knowledge. In time, a portion of these individuals uniquely talented in research were asked to teach the art as well as the science of medicine. The role models they constituted were instrumental in separating the art form and humanity of medicine from the science of medicine. However, in the post-grant era of the late 1970s and 1980s, changes in the economic status of many medical schools forced deans to focus on the pragmatic issues of survival rather than growth. Resolutions were often made at the expense of the conceptual foundations of medical education. The

Address correspondence/reprint requests to Dr. Gilles R.G. Monif, Department of Obstetrics and Gynecology, Creighton University School of Medicine, 601 North 30th Street, Suite 4810, Omaha, NE 68131.

Received February 18, 1994

Accepted July 7, 1994

Commentary results of their decisions further changed the quality of the educational product. As progressively more students' performances failed to meet the threshold standards of credentialing, the standards were changed on the rationale that the volume and the variety of clinical materials were too extensive to expect any novice to fully comprehend or master. The acceptance of such alterations by the academic faculties who were responsible in our system for accountability delayed the perception that there was a crisis in medical education. Today, over 80% of medical deans interviewed acknowledge that there is a crisis.[2] While this acknowledgment has been commonplace, ensuing "solutions" have been relatively piecemeal and, by and large, ineffectual.

### INNOVATIONS AND CURRICULUM CHANGES

Without much introspection, the immediate short-term response in many institutions has been to address the acknowledged educational deficits by changing the curriculum. Without understanding why medical education had been fragmented into nonintegrated unit blocks, medical educators utilized the expansion of factual knowledge to remove the process of synthesis from the central core of medical education. New teaching and testing methodologies friendly to such a thrust were initiated and have remained in effect as students continue to be evaluated primarily through a numbers-crunching process that minimizes faculty individualized evaluation. The end result is an educational system that teaches students how to become depositories of factual information rather than how to ask questions and to develop the ability to make valid observations about the natural situations that they experience.

While there will continue to be adjunctive experiments in curriculums and teaching techniques, the term "new" in describing these manipulations is usually a little out of focus. Reliance on curriculum or technological changes underscores a fundamental lack of an understanding of the education process. The features and talents that constitute a good teacher have changed very little since the time of Aristotle. The curriculum does not make a good teacher; rather, a good teacher makes the curriculum. Most of what is presented as "new" is not new. In most instances, these "innovations" have already failed at a number of institutions. "New" does not necessarily equate "good." Too often, new concepts, like revolutions, tend to destroy the sustaining tenets before the new concepts have been challenged, rechallenged, and determined to be true contributions to the valid body of knowledge or methodology. The Flexner report gave to medicine a successful methodology for medical education that worked in parallel to the scientific method. However, medical educators, not fully understanding the governing tenets set forth in that report, seemed to have failed to safeguard its valid core against the gradual incorporation of concepts that has resulted in a significant alteration of its end product.

One disturbing feature is that, when one reads articles devoted to medical education, innovations in medical education too often have been catalyzed by the availability of foundation monies. The genuine desire to understand the educational process necessary to produce a fine physician has suffered in the hands of project-driven curriculum committees. "New" methodologies have often been regional with limited transplant ability, e.g., Harvard's much-heralded "New Pathway."[1,3] The talent pool at Harvard is not reproducible at most institutions. The success of the small-group concept, which is an outstanding feature of their program, is dependent on the quality of faculty leadership as well as on the talents that individual medical students bring to the group.

### EDUCATION PER SE

One unfortunate by-product of the relegation of medical education to a position of inferiority has been the tendency to obscure what education entails. Donald Barron, a noted educator, once said that education requires learning that is a disciplined pattern of thinking and analysis. "Very often it is not what is taught, but the environment in which it is taught, which becomes the catalyst of true learning." The modern medical student has become an audiovisual reader, assimilating increasingly large amounts of information, often without observing any of the relationships among the facts.[4] Medical educators have failed to give students the overall perspectives necessary to recognize that biological systems are goal related. Failure to achieve such a perception often results in an individual who is inundated by factual information.

The priorities of J. Michael Bishop for teaching were threefold: 1) to inspire, 2) to challenge, and 3) to impart information. To meet these priorities, the educator must teach. The art of teaching is the art of asking questions that, in turn, stimulate the student to perceive the need to learn. A recognition of this need is a primary stimulus for learning. Too many medical educators tend to stress only factual knowledge and, in so doing, clutter the mind so that the student has little time left to think, analyze, decide, and conclude. Huxley once said that the product of true learning is that which is left after all factual knowledge has been forgotten. When one strips factual information from the student, is he or she educated? Does the student have the tools necessary to self-educate? Part of the process of being a good educator is to teach students how to observe. Had Bishop chosen a fourth priority, it might well have been this: Observation is the starting point of all science. An observation is not something to be explained away, but rather something to be clarified as to why it occurred. The process of observation goes beyond the obvious and looks for objective results governed by natural laws. The purpose of observation and ultimately of scientific investigation is the rendering of order out of diversity. Teaching the student to be a sophisticated observer is a function of the basic sciences. Medical education should serve to teach students not to be contractors who can react with limited skills to the factual materials present but rather to be architects who will bring order out of diversity imposed by materials given as facts.

Education is a mutually agreed-upon contract between the educator and student that entails responsibilities on both sides of the coin. While teaching is the responsibility of faculty employed by an educational institution, learning is the responsibility of the student. Failure of the student to accept this responsibility needs a constantly adhered-to structured response on the part of an educational institution to either modify behavior and motivate the student to learn or to terminate the opportunity or privilege to be taught. Failure to learn, just as failure to educate, is a breech of contract.

### A SOLUTION—TOUGH LOVE

Medical educators from deans through faculty ranks do not seem to love their medical students sufficiently. If they did, more "tough love" would be apparent in the nation's medical schools. Unfortunately, many deans, department chairpersons, and full-time faculty members are tangentially involved in the educational business. The funding of medical education, which stresses the teaching of undergraduate medical-school students by professional educators devoid of clinical or research fiscal agendas, has adversely affected the real business of medical schools as they have evolved. Since World War II, medical schools have become primarily health-care delivery and medical-research institutions or franchises for the practice of sophisticated medicine which are partially underwritten by state-federal monies and student tuitions. Today's medical-school deans are less conceptual leaders and progressively more men and women striving to bring fiscal order out of evolving chaos and dwindling resources.

The crisis in medical education is, in good part, a crisis in leadership. Deans need to recognize their relative limitations and delegate trust. An old French adage says that to criticize is easy but to create is difficult. If Ten Commandments were drafted to impact on the progressive deterioration of medical education, they might read along the lines of those listed in Table 1.

If deans cannot abandon their love affair with curriculums, there is a place where innovation may have merit. A neglected facet of medical education is a better utilization of the undergraduate-college prerequisite courses used to prepare and evaluate potential medical-school candidates. More emphasis can and should be placed on what comes in the front door rather than trying to modify a preset individual. Personality strengths are precast prior to entry into graduate studies. Greater focusing is needed on courses that enhance selected students' aptitudes for "high-touch" medicine rather than "high-tech" medicine. Human enrichment courses in medical anthropology, sociology (including health economics), ethics, and philosophy are potential counterbalances to the widening gap between physician and patient and its adverse impact on medicine.[5,6]

### CONCLUSIONS

Academic medicine should not look for solutions to come from the federal government. Whatever is

**TABLE 1. Ten educational commandments for medical-school deans**

| | |
|---|---|
| I | Deans must abide by the concept of the buck-stops-here when it comes to the consequences of mediocrity in medical education. |
| II | Deans must NOT convene another blue-ribbon panel to "study" the current crisis in medical education. |
| III | Deans must read the reports of previous committees that have "studied" this problem. |
| IV | Deans must identify and quantify those monies derived from tuition endowments and from state and federal governments designated for EDUCATION and NOT use these funds for revenue-enhancing projects. |
| V | Deans must strive to identify unique teaching skills on the part of individual faculty or of given divisions within departments and facilitate the students' access to these TEACHERS or TEACHING EXPERIENCES. |
| VI | Deans must increase the number of patients medical students interface with and the quality of these experiences. |
| VII | Deans must designate education as a true priority and demonstrate the depth of this commitment by creating a number of chairs awarded on the basis of teaching excellence. |
| VIII | Deans should allow these chairpersons to usurp partial control of medical education by allowing them to function as implementors, originators, or advocates of the policy that governs the molding and enlightenment of physicians-in-training. |
| IX | Deans must recognize their prowess and insightfulness or relative lack thereof and self-administer or implement anti-hubristic measures. |
| X | Deans should focus their talents and energies on building buildings, engendering fiscal revenues, and enhancing institutional academic prowess and leave education to committed, talented individuals. |

engendered will have to come directly from a handful of courageous and talented deans. These changes should incorporate the dominant concept that systems or institutions do not educate; teachers within these institutions do.

### REFERENCES

1. Tosteson DC: New pathways in general medical education. N Engl J Med 322:234–238, 1990.
2. Cantor FC, Cohen AB, Banker DC, et al.: Medical educators' views on medical education reform. JAMA 265:1002–1006, 1991.
3. Tosteson DC: New pathways for medical education. JAMA 265:1022–1023, 1991.
4. Bloom SW: Structure and ideology in medical education: An analysis of resistance to change. J Health Soc Behav 29:294–306, 1988.
5. Showstach J, Fein O, Ford D, et al.: Health of the public: The academic response. JAMA 267:2497–2500, 1992.
6. Schroeder S, Zones JS, Showstach JA: Academic medicine as a public trust. JAMA 262:803–812, 1989.
